# The effects of mobilization and manipulation on mortality and structure, function and inflammatory markers in cervical blood vessels: a systematic review and meta-analysis of studies in healthy animals and animals with pre-existing vascular pathology

**DOI:** 10.3389/fcvm.2025.1700494

**Published:** 2026-01-05

**Authors:** Rogier F. de Best, Michel W. Coppieters, Christos Bozis, Kirsten A. van Gelderen-Ziesemer, Carlijn R. Hooijmans, Gwendolyne G. M. Scholten-Peeters

**Affiliations:** 1 Faculty of Behavioural and Movement Sciences, Vrije Universiteit Amsterdam, Amsterdam Movement Sciences—Program Musculoskeletal Health, Amsterdam, Netherlands; 2 School of Health Sciences and Social Work, Griffith University, Brisbane and Gold Coast, QLD, Australia; 3 Faculty of Behavioural and Movement Sciences, Vrije Universiteit Amsterdam, Amsterdam, Netherlands; 4 Medical Library, Amsterdam UMC, Vrije Universiteit Amsterdam, Amsterdam, Netherlands; 5 Department of Anesthesiology, Pain and Palliative Care (Meta Research Team), Radboud University Medical Centre, Nijmegen, Netherlands

**Keywords:** hemodynamic, adverse events, dissection, atherosclerosis, carotid artery, manual therapy, systematic review, complications

## Abstract

**Background:**

The safety of cervical spinal manipulative therapy (cSMT) remains debated. Although generally considered safe, cSMT has been linked to rare vascular complications such as cervical arterial dissection and cerebrovascular events. One hypothesis is that pre-existing vascular conditions may increase susceptibility to mechanical forces induced by cSMT, but supporting evidence is limited. Considering ethical and practical constraints in human research, these potential adverse events can only be studied in animal models.

**Aim:**

To evaluate the effects of cSMT on structure, function and inflammatory markers of cervical arteries and mortality in healthy animals and animals with vascular pathology.

**Methods:**

A systematic search of PubMed, Embase.com and EBSCO/CINAHL was conducted on March 19, 2025 to identify controlled experimental studies on the effects of cSMT in animals with and without vascular pathology. Two independent reviewers conducted study selection, risk of bias assessment and data extraction. Random-effects meta-analyses were performed for stenosis rate, cross-sectional area, mechanical properties (load, stress, strain), blood flow parameters (velocity, volume), and inflammatory markers (high sensitivity C-Reactive Protein and macrophages). Hedges *g* was used to report effect sizes.

**Results:**

Six studies met the selection criteria. Most showed unclear risk of bias. In healthy animals, cSMT had no significant effect on stress, strain or blood flow volume (*n* = 2). In animals with vascular pathology, cSMT significantly increased macrophage infiltration (*n* = 2), but had no effect on stenosis rate (*n* = 3), cross-sectional area (*n* = 2), stress (*n* = 2), strain (*n* = 2) or peak systolic velocity (*n* = 2). In animals with vascular pathology compared to healthy animals receiving cSMT, mechanical strain decreased significantly (*n* = 2), while no difference was found for mechanical stress (*n* = 2). No study reported mortality as an outcome.

**Conclusion:**

In animals with vascular pathology, cSMT increases inflammatory responses and atherosclerosis reduces tensile strain of cervical arteries. These findings warrant cautious interpretation and may not translate to humans.

**Systematic Review Registration:**

https://www.crd.york.ac.uk/PROSPERO/view/CRD42023471983, PROSPERO CRD42023471983.

## 1 Introduction

Cervical spine manipulative therapy (cSMT) is a frequently used intervention by physiotherapists aimed at relieving pain and reducing disability in patients experiencing headache and/or neck pain (1–3). Despite its common use, the safety of cSMT remains a topic of debate (4–6). Although cSMT is generally considered safe, it has been identified as a potential risk factor for serious vascular complications, including cervical arterial dissection and cerebrovascular events, such as ischemic stroke (7, 8). Due to their anatomical orientation in the neck, the carotid and vertebral arteries are considered the most susceptible to injury during cSMT (9, 10). Moreover, the carotid artery is a common location for atherosclerosis (11), which is considered a potential risk factor for ischemic stroke, potentially resulting from carotid stenosis or the instability of plaques (12).

To mitigate the risk of vascular complications following cSMT, the International Federation of Orthopaedic Manipulative Physical Therapists (IFOMPT) has developed a framework, referred to as the IFOMPT Framework (13). This framework hypothesizes that pre-existing vascular pathology is a prerequisite for subsequent arterial wall damage induced by mechanical stimuli such as cSMT. Therefore, it is of utmost importance according to the framework that clinicians can identify underlying vascular pathologies that may present with symptoms mimicking musculoskeletal neck pain or headache. Establishing a causal relationship between arterial damage and cSMT in clinical settings, however, remains challenging due to the typically unknown vascular integrity prior to the intervention (14).

To better understand the effects of cSMT on structure, function and inflammation in the cervical arteries, and mortality, animal studies have been conducted. These studies aimed to reveal how cSMT affects vascular integrity, biomechanical properties, hemodynamics and histological features in both healthy arteries and arteries with pre-existing vascular pathology (14, 15). However, a systematic review on this topic is lacking.

A systematic review is necessary to summarize research findings related to their risk of bias and identify any gaps in the current research. Given the ethical and practical constraints that preclude such studies in humans, the use of animal models is still essential. Therefore, this study aimed to summarize the effects of cSMT on the structure, function and inflammatory markers in cervical arteries and mortality in healthy animals, or animals with pre-existing vascular pathology.

## 2 Methods

In this systematic review we addressed three research questions:

1. In healthy animal models, what are the effects of cSMT on vascular structure, function, inflammatory markers or mortality?
2. In animal models with induced cervical vascular pathology, what are the effects of cSMT on vascular structure, function, inflammatory markers or mortality?
3. In animal models treated with cSMT, what is the effect of induced vascular pathology vs. no induced vascular pathology on vascular structure, function, inflammatory markers or mortality?

The methodology was predetermined and preregistered in PROSPERO CRD42023471983 adhering to guidelines for preclinical systematic reviews and meta-analysis. The reporting follows the Preferred Reporting Items for Systematic Reviews and Meta-Analyses (PRISMA 2020) statement (16).

### 2.1 Literature search

A comprehensive search was performed in three bibliographic databases (PubMed, Embase.com and EBSCO/CINAHL) from inception to March 19, 2025 in collaboration with a medical information specialist (KAZ). The search strategy combined Medical Subject Headings (MeSH) and free-text terms, using synonyms and related keywords for “cervical vertebrae” and “musculoskeletal manipulation”. To enhance the comprehensiveness of the search, both forward and backward citation tracking were conducted using Citationchaser (https://estech.shinyapps.io/citationchaser).

The search filter for laboratory animals for different literature databases was used to limit results to laboratory animals (17). No date or language restrictions were applied. Duplicate articles were excluded using Endnote X20.0.1 following the Amsterdam Efficient Deduplication (AED)-method. The full search strategy used for each database is detailed in Supplementary Appendix 1.

### 2.2 Study selection

The study selection process was independently conducted by two researchers (RB, CB) using Rayyan (18). Initial screening involved assessing the potential eligibility of titles and abstracts. Subsequently, the full texts of potentially eligible studies were reviewed to determine whether these met predetermined selection criteria. Any discrepancies in study selection were resolved through discussion between the two researchers. When consensus could not be achieved, a third reviewer (GSP) was consulted to make the final decision.

For the three research questions, studies were eligible for inclusion if they met the following criteria: (1) An original experimental controlled animal study or crossover trial was conducted; (2) Quantified at least one outcome effect of the intervention, such as vascular structure, function, inflammatory markers or mortality. For Research Questions 1 & 2, eligible studies included interventions consisting of any form of cSMT on the cervical spine (e.g., mobilization, manipulation, manual therapy, chiropractic, osteopathic, orthopaedic manipulation), while the control groups either received no intervention or underwent a sham intervention.

For Research Question 1, studies on healthy animals of all species, ages and sexes, without pre-existing cervical vascular pathology were eligible. For Research Question 2, studies on animals of all species, ages and sexes with pre-existing cervical vascular pathology (e.g., cervical arterial dissection, aneurysm, peri-arterial hematoma, atherosclerosis or thrombosis) were eligible. For Research Question 3, studies on animals of all species, ages, and sexes exposed to cSMT on the cervical spine were eligible. The interventions involved an induced vascular pathology (e.g., atherosclerosis, dissection), while the control group did not receive any form of induced vascular pathology. Studies were excluded when they met at least one of the following criteria: (1) the study did not collect new, original data, (2) did not use an *in vivo* animal model, (3) involved other forms of conservative interventions, such as massage, acupressure, transcutaneous electrical nerve stimulation, exercise or myofascial treatment (e.g., dry needling, acupuncture, trigger point release methods), (4) co-interventions were applied, (5) no or incorrect control groups, and (6) no evaluation of vascular structure, function, inflammatory markers or mortality. No studies were excluded based on language or publication date.

### 2.3 Data extraction

Two reviewers (RB and CB) independently extracted the study characteristics and outcome data using a predetermined Excel sheet. The extracted data included: study design, animals (species, sex, age and weight), disease model (lesion characterization, method of induction, and location), intervention (type, dose, intensity, frequency, time between interventions, and time between induced pathology and intervention), control group and type of outcome measures (vascular structure, function, inflammatory markers or mortality). In case of continuous outcomes, means, standard deviations (SD), and number of animals (*n*) were recorded. If standard errors (SE) were reported rather than SD, SE values were converted to SD for consistency.

In case of dichotomous data, the total number of animals and the number of events in both the experimental and control group were extracted. In case of discrepancies, the extracted information was discussed between the two reviewers. If consensus could not be reached, a third reviewer (GSP) was consulted and made the final decision. Authors of the original studies were contacted if data were unclear or not reported. If the authors did not respond after two reminders, a universal desktop ruler (ImageJ, Fiji) was used to extract data from figures by the two reviewers independently (RB and CB).

### 2.4 Risk of bias assessment

Risk of bias was assessed using SYRCLE's Risk of Bias tool by two independent reviewers (RB and CB). This tool comprises ten items that evaluate selection bias, performance bias, detection bias, attrition bias, reporting bias and other types of bias. A ‘yes’ score indicates a low risk of bias, a ‘no’ score indicates a high risk of bias, and a ‘?’ score indicates an unclear risk. To overcome the common issue of judging too many items as “unclear risk of bias” due to poor reporting of essential methodological details, three items were added regarding reporting quality: reporting any method for randomization, blinding at any level, and presence of a sample size calculation. Yes, indicates reported, no indicates not reported. Discrepancies in scoring were resolved through discussion between the two reviewers. If consensus could not be reached, a third researcher (GSP) was consulted to render the final decision. The percentage agreement between the two reviewers was calculated to measure inter-rater agreement.

### 2.5 Syntheses of results

Data were analysed using R software (RStudio 2021.09.0). Meta-analyses were performed depending on homogeneity in the population, type of intervention, and outcome measures between at least two studies. Otherwise, a descriptive summary was provided. Data were organized per research question and outcome measure (i.e., structure, function, inflammatory markers or mortality).

For continuous variables, differences in outcome measures between the intervention group(s) and control groups were calculated as standard mean differences (SMD), and 95% confidence intervals (95%CI) with Hedges *g* correction. For dichotomous variables, risk ratios with 95%CI were reported. For repeated measurements at different time points within a study, the overall effect of the intervention was first presented using the largest recorded effect size, regardless of time, from each study. Data were pooled using a random-effects model. Statistical heterogeneity was assessed and reported using I-squared (*I*^2^).

Subgroup analyses were conducted when at least 10 independent comparisons were available for type of animal, type of lesion on the cervical arteries, type of intervention, and type of outcome. If applicable, depending on the number and type of studies, (post-hoc) publication bias was assessed using trim-and-fill analyses if at least 15 original studies were included.

## 3 Results

### 3.1 Study selection

The literature search yielded a total of 5,456 studies, with 1,156 from PubMed, 1,148 from Embase.com, and 3,152 from EBSCO/CINAHL. Backward citation tracing identified 192 studies, and forward citation tracking yielded 47 studies.

After removing duplicates identified across the multiple databases, 4,508 unique studies were subjected to title and abstract screening. Following this step, 4,499 studies were excluded, leaving 9 studies for full-text review. Of these, 6 studies met the inclusion criteria and were incorporated into the review. Citation tracking did not result in additional included studies.

**Figure 1:**
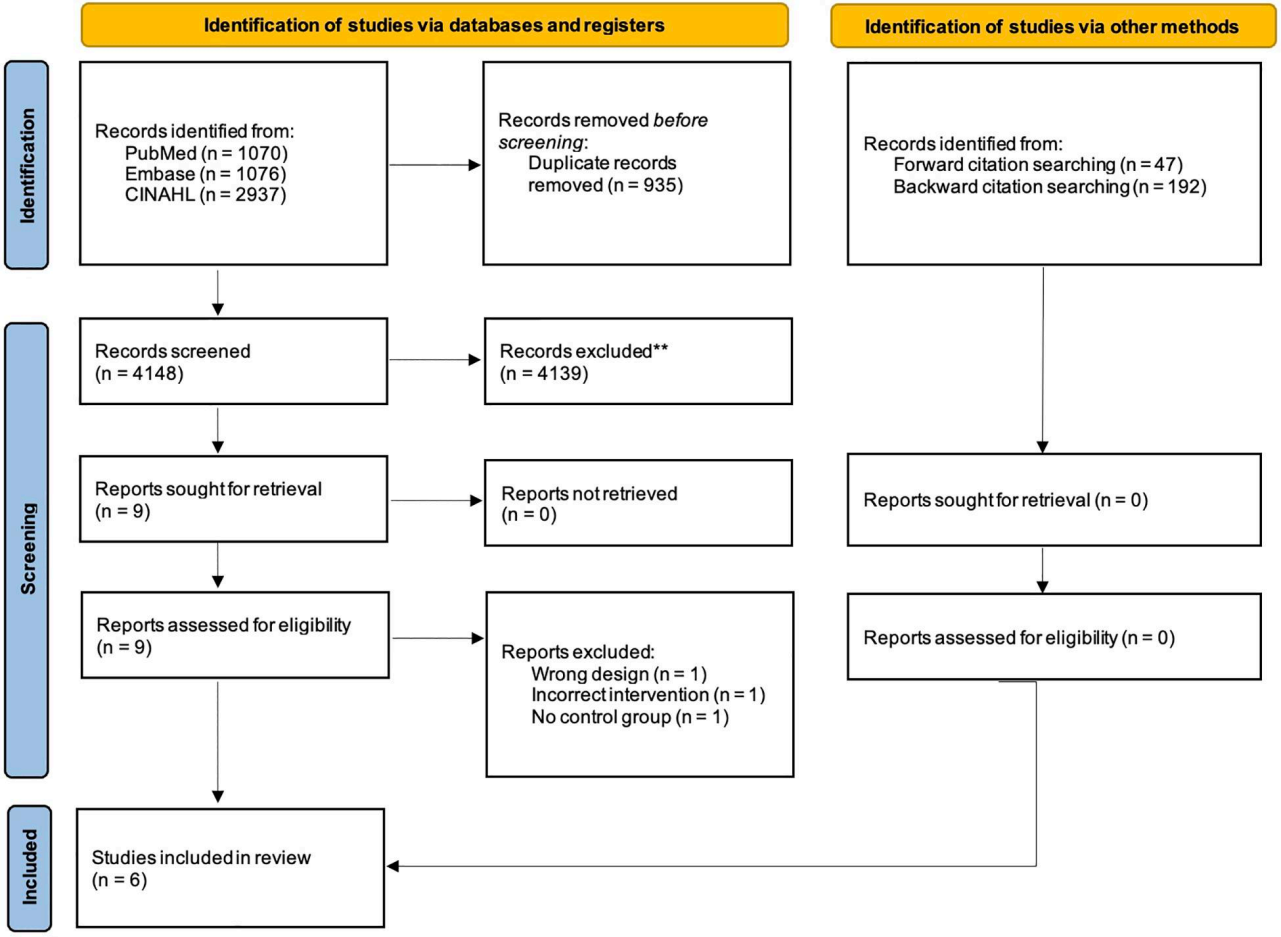
Flowchart of study selection.

The inter-reviewer agreement of the study selection before deliberation was 77.8%. The PRISMA flowchart illustrating the search and selection process is presented in Figure 1.

### 3.2 Study characteristics

Six studies were included in this review: five studies addressed Research Question 1, five studies addressed Research Question 2, and 4 studies addressed Research Question 3. Of the six included studies, four used white rabbits (19–22), one involved cynomolgus monkeys (23), and one study used pigs (15). Five studies included male animals (19–23), while one study included female animals (15). The rabbits were 3 months old (19–22), which corresponds to a juvenile stage in their development. Although this age does not reflect full vascular maturity, the experimental methods used to induce atherosclerosis in these juvenile rabbits, such as balloon catheter injury and high-fat diets, have been validated as effective models for replicating the pathophysiological features of atherosclerosis in humans (24). The age of the pigs and monkeys was not reported (15, 23).

In Research Question 2, all studies investigated disease models of atherosclerosis (19–23). For Research Question 3, the disease models serving as the intervention was atherosclerosis (19–22).

Regarding the application of cSMT across all research Questions, In four studies, cSMT was performed by experienced physiotherapists or manual therapists (19–22), in one study by 2 medical doctors (23) and in one by an experienced chiropractor (15).

For Research Questions 1 & 2, four studies applied high velocity low amplitude manipulations (20–23), while one study applied mobilizations (19). The number of manipulations ranged from 1 to 21 between the five studies, although one study did not report the number of manipulations applied (23).

The treatment period for Research Question 1 & 2 varied from a single session to a treatment period of 3 weeks, while in Research Question 3, the treatment period consistently spanned 13 weeks, defined by the duration of the disease model procedure.

Details of the intervention protocols varied considerably between studies. In one study, 20 cervical mobilisations were applied daily to the left side of the neck over a three-week period (19). In another study, two daily HVLA thrust manipulations were performed in end-range cervical rotation, one to each side, over three weeks, resulting in a total of 42 manipulations (20). One study applied two rotational manipulations per session at approximately 100–110 degrees of cervical rotation (21). In another study, two HVLA thrust manipulations were performed bilaterally per session, repeated five times every three days, resulting in 20 manipulations in total (22). In one study, two bilateral rotational HVLA thrust manipulations were delivered twice a week for three weeks, totalling 24 manipulations (23). Lastly, one study reported two HVLA thrust manipulations applied to the left side at C1–2 and C3–4 in a lateral flexion-rotation direction (15). In none of the studies, the intensity of the manipulations or mobilisations was reported. The characteristics of the studies are summarized in Tables 1–3.

**Table 1:** Study characteristics of the effects of cSMT on vascular structure, function, inflammatory markers or mortality between healthy animals (Research Question 1).

References	Population				Intervention					Control		Outcome
	Species	Sex (M/F)	Age (mo)	Weight (Grams)	*N*	Type	Freq.	Treatment period	*F*	*N*	Comparison	Description
Licht et al. (15)	Pigs	F	NR	45,000	14	HVLA	2	1 session	NR	13	No mp or mob	Function
Qi et al. (19)	Rabbits	M	3	2,000–2,400	10	Mob	420	3 weeks	NR	9	No mp or mob	Structure
Qi et al. (20)	Rabbits	M	3	2,000–2,400	8	HVLA	42	3 weeks	NR	10	No mp or mob	Structure Infl. markers
Zhang et al. (21)	Rabbits	M	3	2,000–2,400	8	HVLA	4	1 session	NR	8	No mp or mob	Structure
Zhang et al. (22)	Rabbits	M	3	2,000–2,400	8	HVLA	20	15 days	NR	8	No mp or mob	Structure Infl. markers

M/F, Male/Female; mo, age in months; Lesion char, lesion characterization; Atheroscl, Atherosclerosis; N, number; HVLA, High Velocity Low Amplitude trust manipulation; Mob, Mobilization; Freq, number of mobilizations or manipulations; NO mp or mob, No manipulation or mobilization; F, applied force; infl. markers, Inflammatory markers out of blood samples; NR, not reported.

**Table 2:** Study characteristics of the effects of cSMT on vascular structure, function, inflammatory markers or mortality between animals with induced vascular pathology (Research Question 2).

References	Population								Intervention					Control		Outcome
	Species	Sex (M/F)	Age (mo)	Weight (Grams)	Artery measured	Lesion char.	Severity	Method of induction	*N*	Type	Freq.	Treatment period	*F*	*N*	Comparison	Description
Guan et al. (23)	Monkeys	M	NR	6,000–7,000	Carotid	Atheroscl.	Mild	Scratched wall with a needle; high fat diet	3	HVLA	24	3 weeks	NR	3	No mp or mob	Structure Function
Qi et al. (19)	Rabbits	M	3	2,000–2,400	Carotid	Atheroscl.	NR	Balloon catheter-induced; injury; high-fat diet	8	Mob	420	3 weeks	NR	8	No mp or mob	Structure
Qi et al. (20)	Rabbits	M	3	2,000–2,400	Carotid	Atheroscl.	Severe	Balloon catheter-induced injury; high-fat diet	10	HVLA	42	3 weeks	NR	9	No mp or mob	Structure Infl. Markers
Zhang et al. (21)	Rabbits	M	3	2,000–2,400	Carotid	Atheroscl.	NR	Balloon catheter-induced injury; high-fat diet	8	HVLA	4	1 session	NR	8	No mp or mob	Structure
Zhang et al. (22)	Rabbits	M	3	2,000–2,400	Carotid	Atheroscl.	NR	Balloon catheter-induced injury; high-fat diet	8	HVLA	20	15 days	NR	8	No mp or mob	Structure Infl. markers

M/F, Male/Female; mo, age in months; Severity, severity of stenosis classified as, Mild (<50%); Moderate (50–69%); Severe (>70%) and Occlusion (100%); N, number; HVLA, High Velocity Low Amplitude trust manipulation; Mob, Mobilization; Freq, number of mobilizations or manipulations; NO mp or mob, No manipulation or mobilization; F, applied force; infl. markers, Inflammatory markers out of blood samples; NR, not reported.

**Table 3:** Study characteristics of the effects of induced vascular pathology vs. no induced vascular pathology when treated with cSMT and on vascular structure, function, inflammatory markers or mortality (Research Question 3).

References	Population								Intervention					Control	Outcome
	Species	Sex (M/F)	Age (mo)	Weight (Grams)	Type (MT)	Freq.	*F*	Treatment period	*N*	Artery measured	Lesion char.	Severity	Method of induction	*N*	Description
Qi et al. (19)2018	Rabbits	M	3	2,000–2,400	Mob	420	NR	3 weeks	10	Carotid	Atheroscl.	NR	Balloon catheter-induced injury; high-fat diet	9	Structure
Qi et al. (20) 2019	Rabbits	M	3	2,000–2,400	HVLA	42	NR	3 weeks	8	Carotid	Atheroscl.	Severe	Balloon catheter-induced injury; high-fat diet	10	Infl. Markers
Zhang et al. (21)2017	Rabbits	M	3	2,000–2,400	HVLA	4	NR	1 session	8	Carotid	Atheroscl.	NR	Balloon catheter-induced injury; high-fat diet	8	Structure
Zhang et al. (22)2022	Rabbits	M	3	2,000–2,400	HVLA	20	NR	15 days	8	Carotid	Atheroscl.	NR	Balloon catheter-induced injury; high-fat diet	8	Structure

M/F, Male/Female; mo, age in months; MT, Manual intervention; HVLA, High Velocity Low Amplitude trust manipulation; Mob, Mobilization; Freq, number of mobilizations or manipulations; F, applied force; N, number; Lesion char, lesion characterization; Atheroscl, Atherosclerosis; Severity, severity of stenosis classified as Mild (<50%); Moderate (50–69%); Severe (>70%) and Occlusion (100%); infl. markers, Inflammatory markers out of blood samples; NR, not reported.

### 3.3 Risk of bias assessment

Overall, 81.7% of all domains were classified as “unclear” due to the lack of reporting of essential methodological details. In 7 out of the 10 domains, all studies scored “unclear” on similar baseline characteristics, allocation concealment, random housing, random animal selection for outcome assessment, blinding of outcome assessor, free of selective outcome reporting, and free of other sources of bias. All studies scored a high risk of bias in the domains of blinding of caregivers and investigators, as none of the studies reported the use of blinding procedures. Two studies adequately reported an allocation sequence (20, 21) and two studies adequately addressed incomplete data (22, 23).

**Figure 2:**
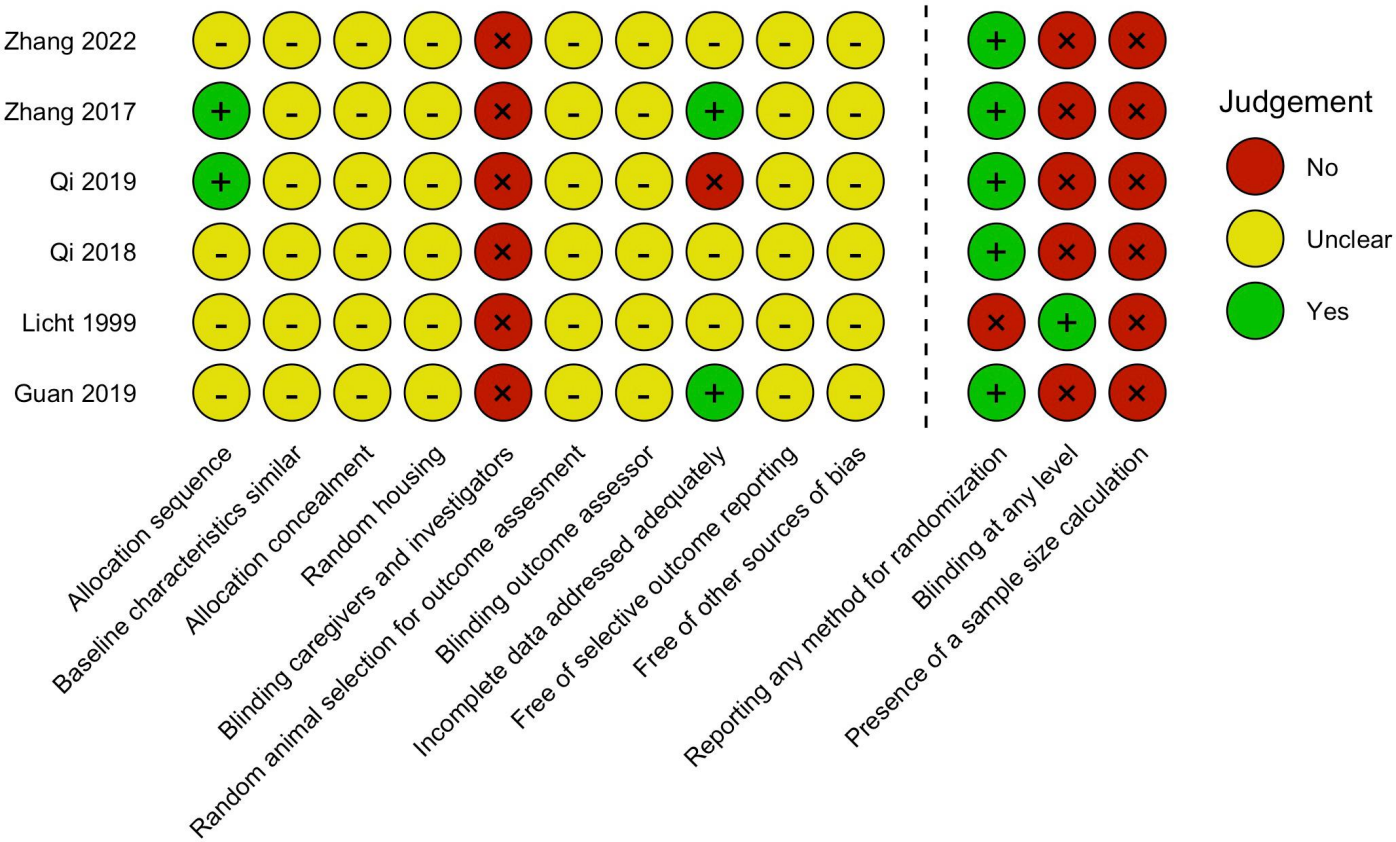
Risk of bias assessment*.* Items 1–10 assessed risk of bias, with “yes” indicating low risk of bias, “no” high risk of bias, and “-” unclear risk of bias. Items 11–13 assess study quality by scoring reporting, a “yes” score indicates reported and a “no” score indicates unreported.

Because of the poor reporting of essential details, we added three reporting quality questions. Randomization at least at one level was reported in 83.3% of the studies. Only one study reported blinding at least at one level (i.e., outcome assessment) (15). and none of the studies reported a sample size calculation. The inter-reviewer agreement for the risk of bias assessment was 93.6%. The results of the risk of bias assessment are presented in Figure 2.

### 3.4 Results of syntheses

Meta-analyses were conducted for all three Research Questions. The outcomes for Research Question 1 were: mechanical properties (stress and strain), and blood flow (volume). Regarding Research Question 2, meta-analyses were performed for stenosis rate, cross-sectional area, mechanical properties (stress and strain), blood flow (Peak Systolic Velocity), and the number of macrophages. Regarding Research Question 3, meta-analyses were performed for two outcome measures namely mechanical properties related to stress and strain.

**Figure 3:**
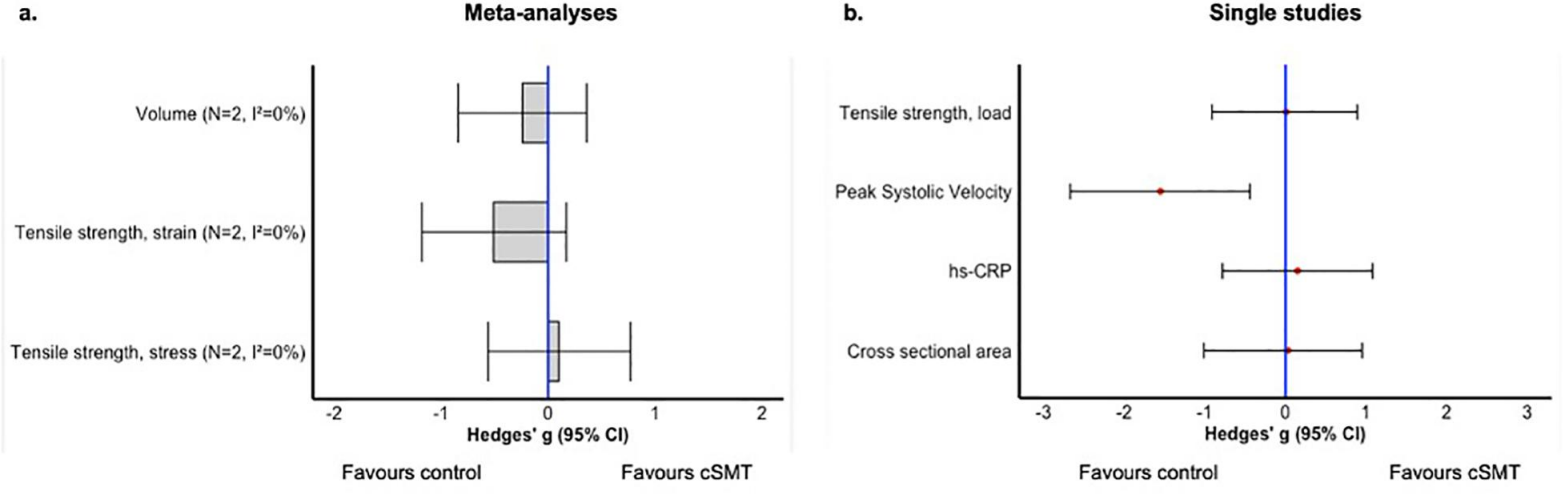
Overview of the effects of cSMT between healthy animals (research question 1); meta-analyses **(a)** and single study results **(b)**. *N*, number of included studies; NA, not applicable.

**Figure 4:**
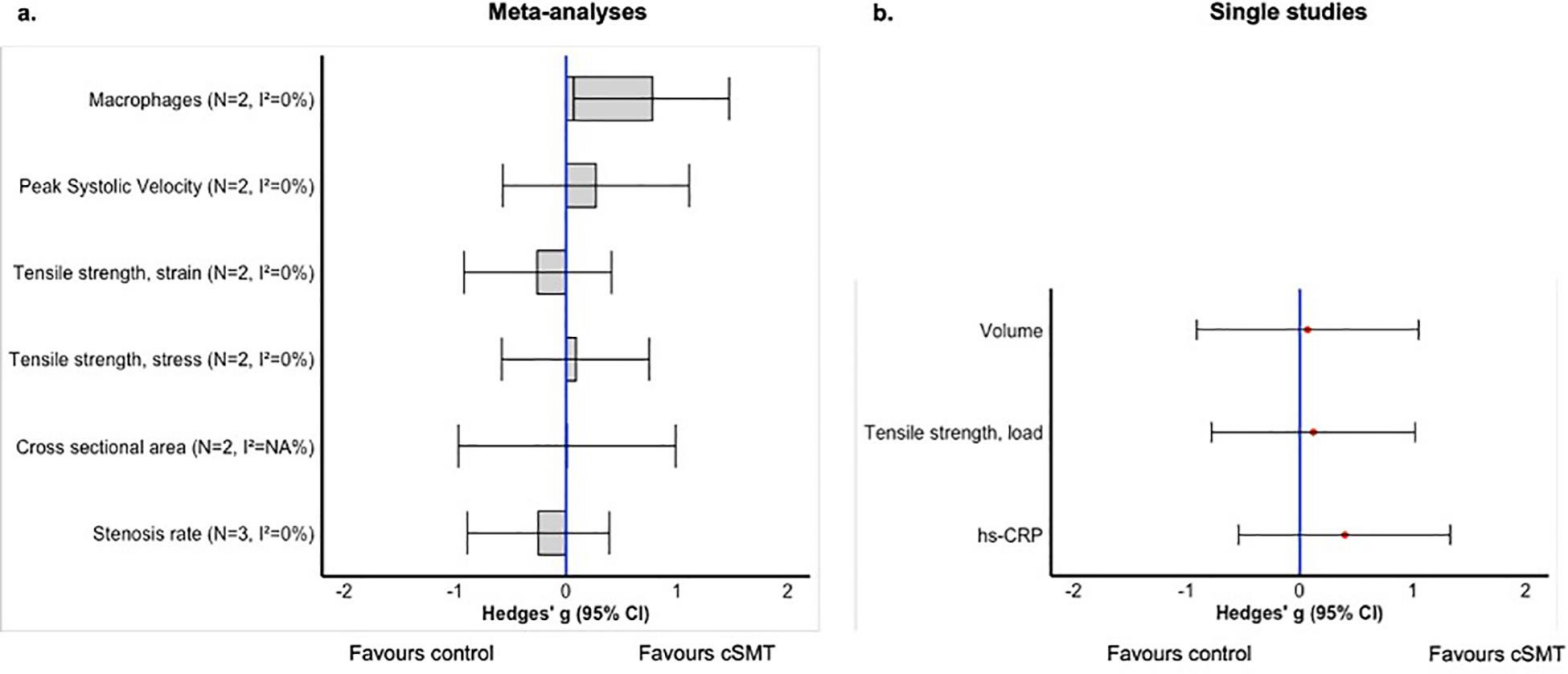
Overview of the effects of cSMT between animals with induced vascular pathology (research question 2): meta-analyses **(a)** and single study results **(b)**. *N*, number of included studies; NA, not applicable.

**Figure 5:**
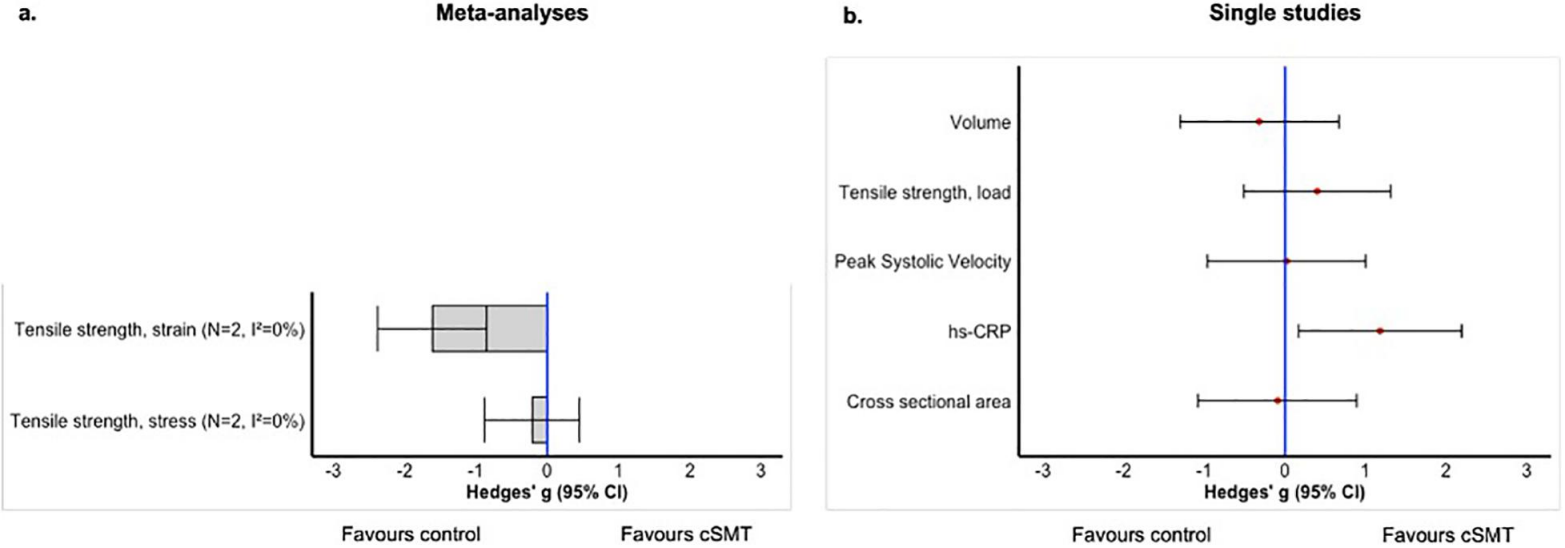
Overview of the effects of induced vascular pathology vs. no induced vascular pathology when treated with cSMT (Research Question 3); meta-analyses **(a)** and single study results **(b)**. *N*, number of included studies; NA, not applicable.

An overview of the results of the meta-analyses and single studies, categorized by research question, is presented in Figures 3–5. A comprehensive overview of all forest plots, categorized by research question, is provided in Supplementary Appendix 2.

#### 3.4.1 Results of meta analyses

Effects of cSMT compared to no treatment or sham cSMT on structure, function, inflammatory markers, or mortality in healthy animals (Research Question 1).

##### 3.4.1.1 Mechanical property related to stress

Two studies (19, 21) containing 2 independent comparisons, revealed no significant effect of cSMT on stress properties of the carotid artery in healthy rabbits [pooled data, Hedges *g*: 0.10 (−0.56 to 0.77), *n* = 2, *I*^2^ = 0.0%].

##### 3.4.1.2 Mechanical property related to strain

Two studies (19, 21) containing 2 independent comparisons, showed no significant differences in strain properties of healthy carotid artery in rabbits with the meta-analysis including 2 independent comparisons [pooled data, Hedges *g*: 0.51 (−1.18 to 0.17), *n* = 2, *I*^2^ = 0.0%].

##### 3.4.1.3 Blood flow, volume

Two studies (15, 22) containing 2 independent comparisons, investigated the effect of cSMT on the volume of blood flow in healthy carotid artery. The results indicated no significant effect when compared to the control group [Hedges *g*: 0.24 (−0.84–0.36), *n* = 2, *I*^2^ = 0.0%].

#### 3.4.2 Results of single study outcomes

Effects of cSMT on structure, function, inflammatory markers, or mortality between animals with no induced vascular pathology (Research Questions 1).

Data from single studies evaluating the effects of cSMT on various outcome parameters in healthy animals are presented in this section. One study (22) conducted a study with eight rabbits to assess the impact of cSMT on cross-sectional area and found no significant effect [Hedges *g*: 0.03 (−1.01 to 0.89)]. In contrast, the same study (22) also assessed the velocity of blood flow in healthy carotid arteries and observed a significant increase of cSMT on blood flow velocity. [Hedges *g*: −1.55 (−2.67 to 0.44)].

In another study (19), the mechanical properties related to load in the carotid artery were examined in ten rabbits. No significant effect of cSMT on load properties was found [Hedges *g*: −0.01 (−0.91 to 0.89), *n* = 9]. Lastly, one study (20) evaluated the effect of cSMT on inflammatory markers, specifically Hs-CRP levels, in the carotid arteries of eight rabbits. This study found no significant effect of cSMT on Hs-CRP levels [Hedges *g*: 0.15 (−0.78 to 1.08)].

#### 3.4.3 Results of meta analyses

Effects of cSMT on structure, function, inflammatory markers, or mortality between animals with induced vascular pathology (Research Question 2).

##### 3.4.3.1 Stenosis rate

Three studies (20, 21, 23) containing 3 independent comparisons, were included in the meta-analyses. In the carotid artery of monkeys and rabbits, cSMT did not significantly increase the stenosis rate compared to the control groups [pooled data, Hedges *g*: −0.25 (−0.89 to 0.39), *n* = 3, *I*^2^ = 0.0%].

##### 3.4.3.2 Cross-sectional area

Two studies (22, 23) containing 2 independent comparisons (i.e., monkeys and rabbits) were included in the meta-analysis. The results revealed no significant effect of cSMT on the cross-sectional area of the atherosclerotic carotid artery in animal models [pooled data, Hedges *g*: 0.01 (−0.97 to 0.99), *n* = 2, *I*^2^ = NA].

##### 3.4.3.3 Mechanical property related to stress

Two studies (19, 21) containing 2 independent comparisons were included in the meta-analysis. Meta-analysis revealed no significant effect of cSMT on stress properties of atherosclerotic carotid artery in rabbits [pooled data, Hedges *g*: 0.09 (−0.58 to 0.75), *n* = 2, *I*^2^ = 0.0%].

##### 3.4.3.4 Mechanical property related to strain

Two studies (19, 21) containing 2 independent comparisons were included in the meta-analysis, which yielded no significant effect [pooled data, Hedges *g*: 0.26 (−0.92 to 0.41), *n* = 2, *I*^2^ = 0.0%].

##### 3.4.3.5 Blood flow, velocity

Two studies (22, 23) containing 2 independent comparisons investigated the effect of cSMT on the velocity of blood flow in atherosclerotic carotid arteries (i.e., monkeys and rabbits). The meta-analysis revealed no significant effect when compared to the control group [pooled data, Hedges *g*: 0.27 (−0.57 to 1.11), *n* = 2, *I*^2^ = 0.0%].

##### 3.4.3.6 Inflammatory markers, number of macrophages

Two studies (20, 22) containing 2 independent comparisons investigated the effect of cSMT on macrophage levels in the atherosclerotic carotid artery in rabbits. The results indicated a significant effect on the increase of macrophages compared to the control group [pooled data, Hedges *g*: 0.77 (0.07 to 1.47), *n* = 2, *I*^2^ = 0.0%].

#### 3.4.4 Results of single study outcomes

Effects of cSMT on structure, function, inflammatory markers, or mortality between animals with induced vascular pathology (Research Question 2).

One study (19) involving 9 rabbits reported no significant effect of cSMT on load properties of atherosclerotic carotid arteries [Hedges *g*: 0.12 (−0.78 to 1.02)]. Another study (22) assessed the effect of cSMT on the volume of blood flow in atherosclerotic carotid arteries of eight rabbits and found no significant effect when compared to rabbits not receiving cSMT [Hedges *g*: 0.07 (−0.91 to 1.05)]. Additionally, one study (20) evaluated the effect of cSMT and vascular pathology on Hs-CRP levels in carotid arteries of eight rabbits, and found no significant effect of cSMT on Hs-CRP levels in rabbits with atherosclerotic carotid arteries [Hedges *g*: 0.40 (−0.54 to 1.33)].

#### 3.4.5 Results of meta analyses

Effects of induced vascular pathology vs. no induced vascular pathology when treated with cSMT on structure, function, inflammatory markers, or mortality (Research Question 3).

##### 3.4.5.1 Mechanical property related to stress

Two studies (19, 21), with 2 independent comparisons, revealed no significant effect of atherosclerosis on stress properties of the carotid artery rabbits, which all underwent cSMT [pooled data, Hedges *g*: −0.21 (−0.88 to 0.45), *n* = 2, *I*^2^ = 0.0%].

##### 3.4.5.2 Mechanical property related to strain

Two studies (19, 21), with 2 independent comparisons, investigated the effect of atherosclerosis on strain properties of carotid arteries in rabbits receiving cSMT. The strain properties of carotid arteries were significantly lower when compared to rabbits receiving cSMT without vascular pathology [Hedges *g*: −1.61 (−2.38 to 0.85), *n* = 2, *I*^2^ = 0.0%].

#### 3.4.6 Results of single study outcomes

Effects of induced vascular pathology vs. no induced vascular pathology when treated with cSMT on structure, function, inflammatory markers, or mortality (Research Question 3).

One study (22) involving eight rabbits investigated the influence of vascular pathology on the cross-sectional area in rabbits subjected to cSMT. The findings demonstrated no significant effect [Hedges *g*: −0.09 (−1.08 to 0.89)]. In the same study, the velocity of blood flow in atherosclerotic carotid arteries in rabbits receiving cSMT was assessed. No significant difference in velocity was found when compared to rabbits without vascular pathology who also received cSMT [Hedges *g*: 0.02 (−0.96 to 1.00)]. Furthermore, the volume of blood flow in atherosclerotic carotid arteries in rabbits subjected to cSMT was not significantly different from that in rabbits without vascular pathology who also received cSMT [Hedges *g*: 0.32 (−1.30 to 0.67)]. In a study involving eight rabbits assessing the influence of induced atherosclerosis on hs-CRP levels in rabbits receiving cSMT (20), hs-CRP levels were increased significantly in rabbits with induced atherosclerosis when compared to the control group [Hedges *g*: 1.18 (0.17–2.19)]. Lastly, a study with ten rabbits (19) found no significant effect of atherosclerosis on the load properties of the carotid artery in rabbits subjected to cSMT. [Hedges *g*: 0.40 (−0.51 to 1.31)].

#### 3.4.7 Mortality

None of the included studies for the three research questions reported mortality as an outcome. One study (20) reported a single mortality case, where 1 out of 8 (11%) white rabbits with carotid atherosclerosis due to a balloon catheter-induced injury and high-fat diet, died due to diarrhea during the intervention. In contrast, no mortality was observed in the group without carotid atherosclerosis during the same intervention.

### 3.5 Sensitivity analysis

A sensitivity analysis was performed to address potential uncertainties arising from unclear reporting of measures of dispersion. In three articles (19, 21, 22), the variability measure provided referred to a standard deviation (SD) or a standard error (SE) was not clearly reported, and the authors did not respond to our request regarding this. To evaluate the robustness of our findings, we conducted sensitivity analyses by recalculating the effect sizes under two assumptions: first, that the reported values represented standard deviations (SDs), and second, that they represented standard errors (SEs). A comprehensive overview of the forest plots from the sensitivity analyses is provided in Supplementary Appendix 3. The sensitivity analyses confirmed that the results remained robust, supporting the stability and reliability of our findings.

### 3.6 Subgroup analyses and publication bias

Due to the limited number of included studies, subgroup analyses and evaluation of publication bias were not feasible.

## 4 Discussion

This systematic review summarises the findings of six studies that examined the effects of cSMT on vascular structure, function, inflammatory markers and mortality in healthy animal models (Research Question 1), the effects of cSMT on animal models with induced vascular pathology (Research Question 2) and the effect of induced vascular pathology vs. no induced vascular pathology on structure, function, inflammatory markers or mortality in animal models when treated with cSMT (Research Question 3).

Regarding the effect of cSMT in healthy animals (Research Question 1), meta-analyses revealed no significant differences in tensile stress, tensile strain, or blood flow volume between groups. Meta-analyses evaluating the effect of cSMT in animals with induced vascular pathology (Research Question 2) demonstrated an increase in the number of macrophages in cervical vascular arteries, suggesting an enhanced inflammatory response following cSMT. The high frequency of cSMT often delivered bilaterally on a daily basis over several weeks may have contributed to the observed inflammatory and macrophage-related responses. As this frequency exceeds typical clinical practice, it may limit the extrapolation of the results to humans. In addition, some animals exhibited severe atherosclerosis, with carotid stenosis rates of 70%–98% or complete occlusion. Furthermore, one study reported 100–110° of cervical rotation prior to cSMT, applied at end-range, which exceeds the typical range used in human cervical procedures (21). These differences including high-frequency exposure, end-range positioning, and severe underlying pathology, may have amplified the mechanical load on vascular structures and contributed to the observed inflammatory findings. Moreover, these lesion and intervention characteristics reduce the extrapolation of the findings to humans.

Meta-analyses examining the impact of vascular pathology on animals treated with cSMT (Research Question 3) showed a significant reduction in tensile strain of the carotid artery. These findings suggest that pre-existing vascular pathology may increase susceptibility to arterial wall damage and potential complications following mechanical stimuli such as cSMT, supporting the hypothesis outlined in the IFOMPT framework.

These results may indicate that individuals with vascular vulnerability are at greater risk of complications following cSMT. Although the animal findings are not directly translatable to humans, they highlight the importance of thorough vascular screening and judicious clinical decision-making, particularly in patients with cardiovascular risk factors. Nonetheless, these results should be interpreted with caution due to the limited number of studies included in the meta-analyses. Additionally, the observed reduction in mechanical strain was not reported in the investigation for Research Question 2. Other outcomes assessing the effects of vascular pathology, such as cross-sectional area, blood flow parameters (velocity and volume), and tensile load of the carotid artery did not show significant effects.

Some results from individual studies suggested a transient increase in peak systolic velocity following cSMT in healthy animals (Research Question 1), with values returning to baseline within minutes. Additionally, one study reported a significant increase in hs-CRP levels following cSMT in animals with induced vascular pathology compared to healthy controls (Research Question 3). However, these findings stem from single studies and should therefore be interpreted with caution. All included studies in this systematic review focused exclusively on atherosclerosis as the vascular condition, whereas the literature also identifies cervical arterial dissection as a potential complication (4). Consequently, the effect of cSMT on arterial dissection and stroke could not be evaluated.

## 5 Limitations

This systematic review included only six papers regarding the effects of cSMT on structure, function and inflammatory markers of cervical arteries and mortality in healthy animals and animals with pre-existing vascular pathology. The limited number of available studies led to imprecise meta-analytic estimates, hereby reducing the overall certainty of the evidence. In addition, most studies appeared to have an unclear risk of bias due to poor reporting of essential methodological details. The lack of critical methodological information raises significant concerns regarding potential bias in the data and the distortion of results, which could undermine the accuracy of the conclusions drawn from these animal studies.

Although the inclusion of a formal certainty-of-evidence summary (e.g., GRADE) could enhance interpretability, no validated and operational framework currently exists for preclinical animal studies. We therefore applied conceptual principles to address key domains such as risk of bias and heterogeneity narratively rather than through tabulated scoring, in line with the current methodological state of the field (25).

Furthermore, the between study heterogeneity appeared to be high for most analyses. This may be a consequence of the low number of studies included in this systematic review. However, we tried to take this between study heterogeneity into account by using the random effects model. Subgroup analyses to explore causes for this heterogeneity unfortunately could not be conducted due to the low number of studies.

Although atherosclerotic lesions in rabbits, monkeys and pigs resemble those observed in humans, interspecies physiological differences limit the translational applicability of these animal-based findings (24). Moreover, it remains unclear whether the animals developed symptoms associated with the induced vascular pathology. In contrast, humans frequently report symptoms such as headache, dizziness and visual disturbances, which are clinically relevant in the assessment of vascular complication risk (26).

Moreover, details regarding the delivery characteristics of the interventions, such as applied force, direction, velocity, or degree of rotation, were often not reported. This lack of quantification hampers reproducibility, and complicates interpretation of potential mechanical effects. Finally, the majority of included studies came from a single laboratory. While this may ensure procedural consistency, it limits independent validation and raises the possibility of systematic bias. Replication by different laboratories, is therefore needed to strengthen the generalizability of the findings.

## 6 Conclusion and future recommendations

Our findings showed that cSMT significantly increases the number of macrophages in animals with pre-existing vascular pathology. Additionally, animals with atherosclerosis demonstrated reduced vascular tensile strain in the carotid artery compared to healthy controls. However, these findings should be interpreted with great caution due to the limited number of studies included in the meta-analyses, the absence of significant effects on other outcomes in animals with vascular pathology, and study limitations such as a high risk of bias and substantial heterogeneity.

To improve the quality and transparency of future research, adherence to established reporting standards such as the ARRIVE guidelines (27) is strongly recommended. The EQIPD framework (28) may serve as a valuable resource for clinical researchers in selecting appropriate guidelines for improving study quality and reporting (28).

More research is needed to better understand the effects of cSMT in healthy animals and animals with vascular pathology. Ideally, future studies should include larger sample sizes of both male and female animals, appropriate randomization, more details on intervention characteristics, blinded outcome assessment, and use advanced, non-invasive methods where possible. Dedicated animal studies should also investigate the effects of cSMT in the presence of cervical arterial dissection models, given its hypothesized role in increasing the risk of vascular complications. Stronger methodological rigor in animal research remains needed. Transparent reporting of baseline comparability and sample size calculations is also essential to reduce bias and improve reproducibility.

## Data Availability

The original contributions presented in the study are included in the article/Supplementary Material, further inquiries can be directed to the corresponding author/s.
