# Supplementary material for: The effects of mobilization and manipulation on mortality and structure, function and inflammatory markers in cervical blood vessels: a systematic review and meta-analysis of studies in healthy animals and animals with pre-existing vascular pathology
Source: Front Cardiovasc Med. 2026 Jan 5;12:1700494. doi: 10.3389/fcvm.2025.1700494 (PMC12812544; doi:10.3389/fcvm.2025.1700494)
Supplement: Supplementary file 1 [file Table1.docx]

# Appendix 1

# Search strategy

**PubMed Results (19 March 2025)**

| **Search** | **PubMed Query – March 19, 2025** | **Results** |
| --- | --- | --- |
| #4 | #1 AND #2 AND #3 | 1,156 |
| #3 | van der Mierden S, Hooijmans CR, Tillema AH, Rehn S, Bleich A, Leenaars CH. Laboratory animals search filter for different literature databases: PubMed, Embase, Web of Science and PsycINFO. Lab Anim. 2022 Jun;56(3):279-286. doi: 10.1177/00236772211045485. Epub 2021 Sep 24. PMID: 34559023; PMCID: PMC9194806. | 8,332,796 |
| #2 | ("Cervical Vertebrae"[Mesh] OR "Neck"[Mesh] OR cervical[tiab] OR neck[tiab] OR vertebra*[tiab] OR thoracic[tiab] OR cervicothoracic[tiab]) | 898,072 |
| #1 | ("Musculoskeletal Manipulations "[Mesh:noexp] OR "Manipulation, Chiropractic "[Mesh:noexp] OR "Manipulation, Osteopathic "[Mesh:noexp] OR "Manipulation, Spinal "[Mesh:noexp] OR "Manipulation, Orthopedic "[Mesh:noexp] OR (manipulation-therap*[tiab] OR manipulation-treatment*[tiab] OR manipulative[tiab] OR manual-therap*[tiab] OR manual-treatment*[tiab] OR chiropractic*[tiab] OR osteopathic* OR mobilis*[tiab] OR mobiliz*[tiab] OR thrust[tiab])) | 156,062 |

**Embase.com Results (19 March 2025)**

| **Search** | **Embase Query – March 19, 2025** | **Results** |
| --- | --- | --- |
| #5 | #4 NOT 'conference abstract'/it | 1,148 |
| #4 | #1 AND #2 AND #3 | 1,440 |
| #3 | van der Mierden S, Hooijmans CR, Tillema AH, Rehn S, Bleich A, Leenaars CH. Laboratory animals search filter for different literature databases: PubMed, Embase, Web of Science and PsycINFO. Lab Anim. 2022 Jun;56(3):279-286. doi: 10.1177/00236772211045485. Epub 2021 Sep 24. PMID: 34559023; PMCID: PMC9194806. | 10,003,228 |
| #2 | 'cervical vertebra'/exp OR 'neck'/exp OR cervical:ti,ab,kw OR neck:ti,ab,kw OR thoracic:ti,ab,kw OR vertebra*:ti,ab,kw OR cervicothoracic:ti,ab,kw | 1,235,213 |
| #1 | 'musculoskeletal manipulation'/exp OR 'manipulation therap*':ti,ab,kw OR 'manipulation treatment*':ti,ab,kw OR manipulative:ti,ab,kw OR 'manual therap*':ti,ab,kw OR 'manual treatment*':ti,ab,kw OR chiropractic:ti,ab,kw OR osteopathic*:ti,ab,kw OR mobilis*:ti,ab,kw OR mobiliz*:ti,ab,kw OR thrust:ti,ab,kw | 168,034 |

**EBSCO/CINAHL Results (19 March 2025)**

| **Search** | **CINAHL Query – March 19, 2025** | **Results** |
| --- | --- | --- |
| #4 | S1 AND S2 AND S3 | 3,152 |
| #3 | Translated to EBSCO/CINAHL from: van der Mierden S, Hooijmans CR, Tillema AH, Rehn S, Bleich A, Leenaars CH. Laboratory animals search filter for different literature databases: PubMed, Embase, Web of Science and PsycINFO. Lab Anim. 2022 Jun;56(3):279-286. doi: 10.1177/00236772211045485. Epub 2021 Sep 24. PMID: 34559023; PMCID: PMC9194806. | 3,296,506 |
| #2 | ( (MH "Cervical Vertebrae+") OR (MH "Neck+") ) OR TI ( cervical OR neck OR thoracic OR vertebra* OR cervicothoracic ) OR AB ( cervical OR neck OR thoracic OR vertebra* OR cervicothoracic ) OR SU ( cervical OR neck OR thoracic OR vertebra* OR cervicothoracic ) | 221,621 |
| #1 | ( (MH "Manipulation, Orthopedic") OR (MH "Manipulation, Chiropractic") OR (MH "Manipulation, Osteopathic") OR (MH "Manual Therapy") ) OR TI ( ((manipulation-therap* OR manipulation-treatment* OR manipulative OR manual-therap* OR manual-treatment* OR chiropractic OR osteopathic* OR mobilis* OR mobiliz* OR thrust OR high-velocity-low-amplitude) ) OR AB ( ((manipulation-therap* OR manipulation-treatment* OR manipulative OR manual-therap* OR manual-treatment* OR chiropractic OR osteopathic* OR mobilis* OR mobiliz* OR thrust OR high-velocity-low-amplitude) ) OR SU ( ((manipulation-therap* OR manipulation-treatment* OR manipulative OR manual-therap* OR manual-treatment* OR chiropractic OR osteopathic* OR mobilis* OR mobiliz* OR thrust OR high-velocity-low-amplitude) ) | 62,515 |
