# Supplementary material for: The effects of mobilization and manipulation on mortality and structure, function and inflammatory markers in cervical blood vessels: a systematic review and meta-analysis of studies in healthy animals and animals with pre-existing vascular pathology
Source: Front Cardiovasc Med. 2026 Jan 5;12:1700494. doi: 10.3389/fcvm.2025.1700494 (PMC12812544; doi:10.3389/fcvm.2025.1700494)
Supplement: Supplementary file 3 [file Table3.docx]

# Appendix 3.

**Overview of the forest plots from the sensitivity analyses**

Forest plots of the effects of cSMT on vascular structure, function, inflammatory markers or mortality between healthy animals (Research Question 1)

| Structure |
| --- |
| Tensile strength, stress |
| 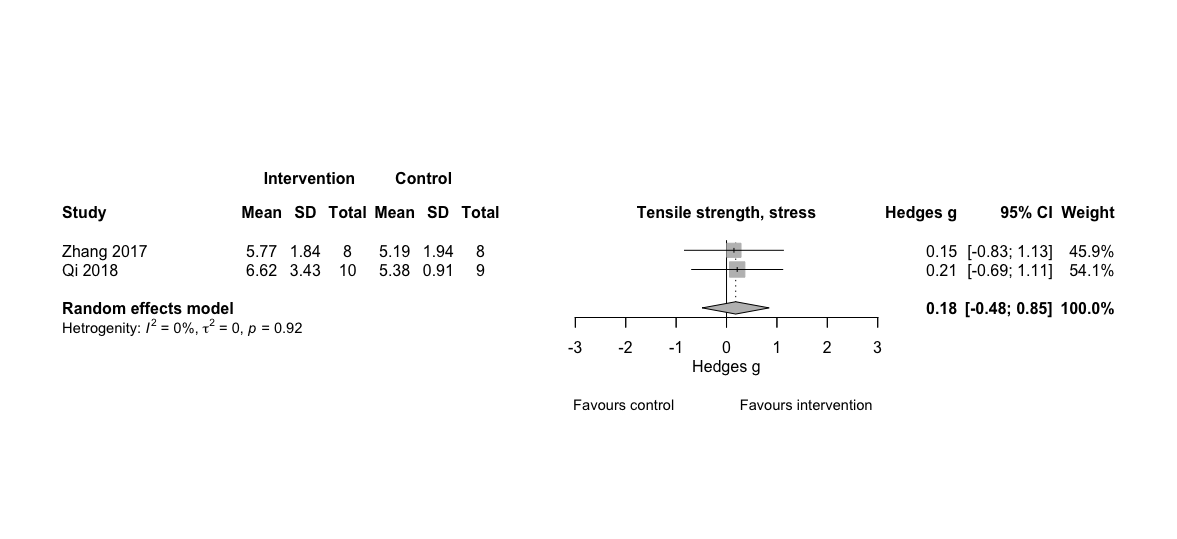 |
| Tensile strength, strain |
| 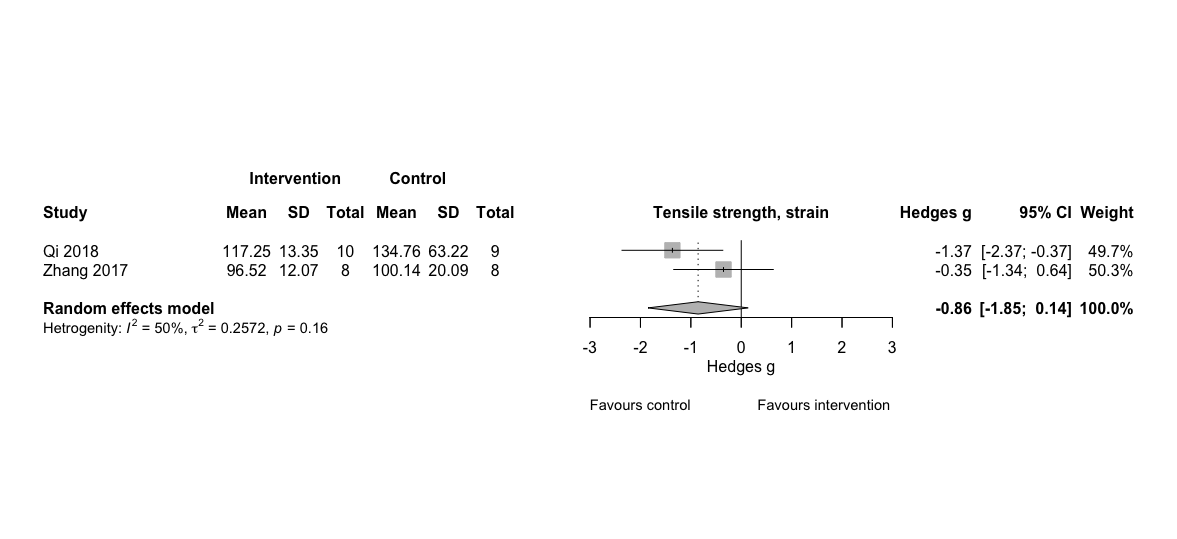 |
| Function |
| Blood flow, Volume |
| **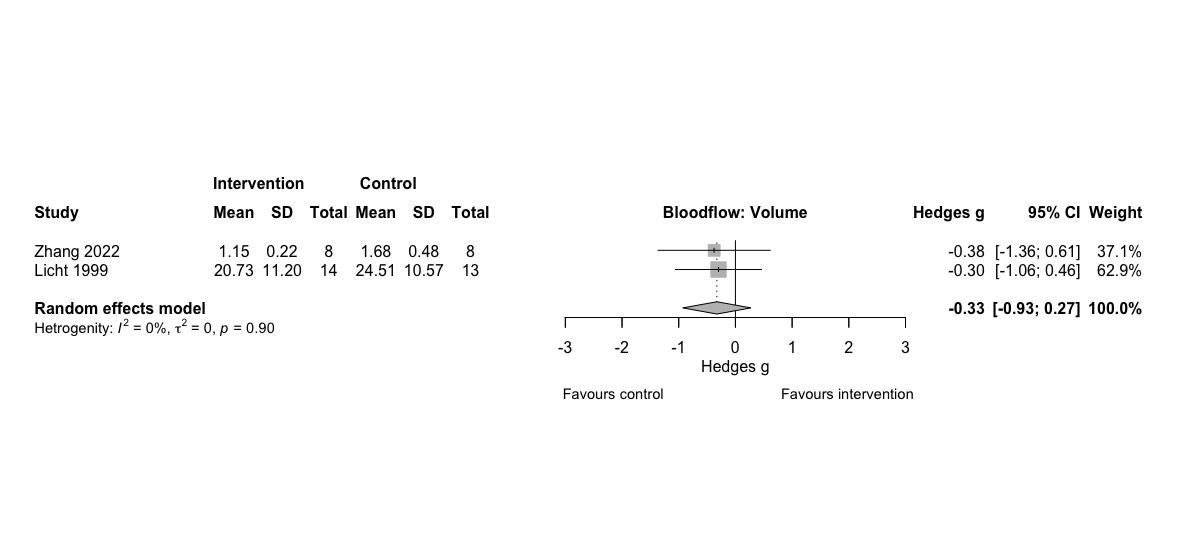** |

Forest plots (Sensitivity analyses) of the effects of cSMT on vascular structure, function, inflammatory markers or mortality between animals with induced vascular pathology (Research Question 2)

| Structure |
| --- |
| Stenosis rate |
| **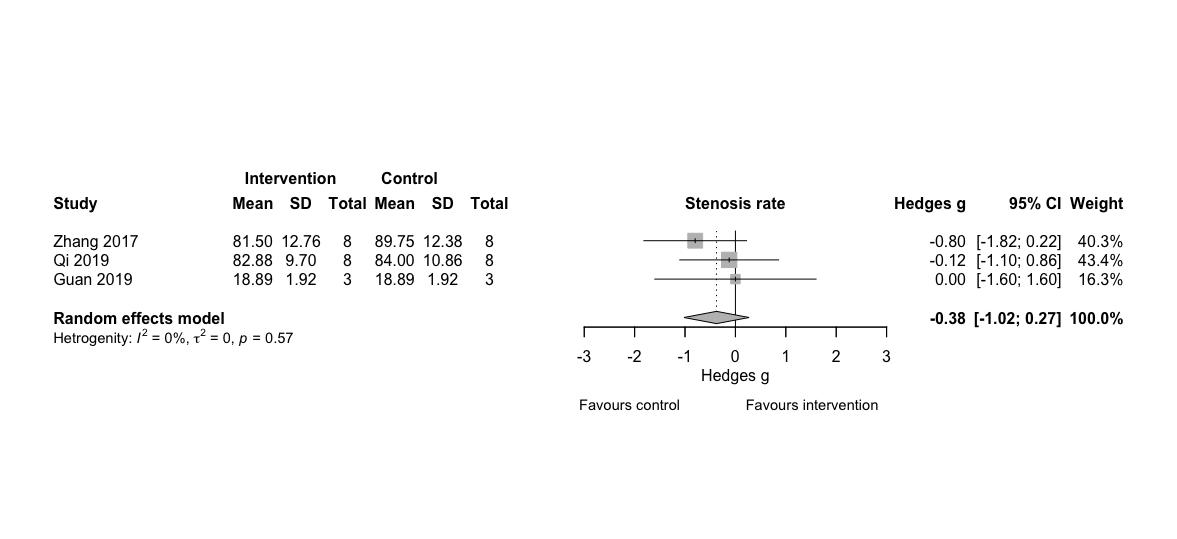** |
| Cross sectional area |
| 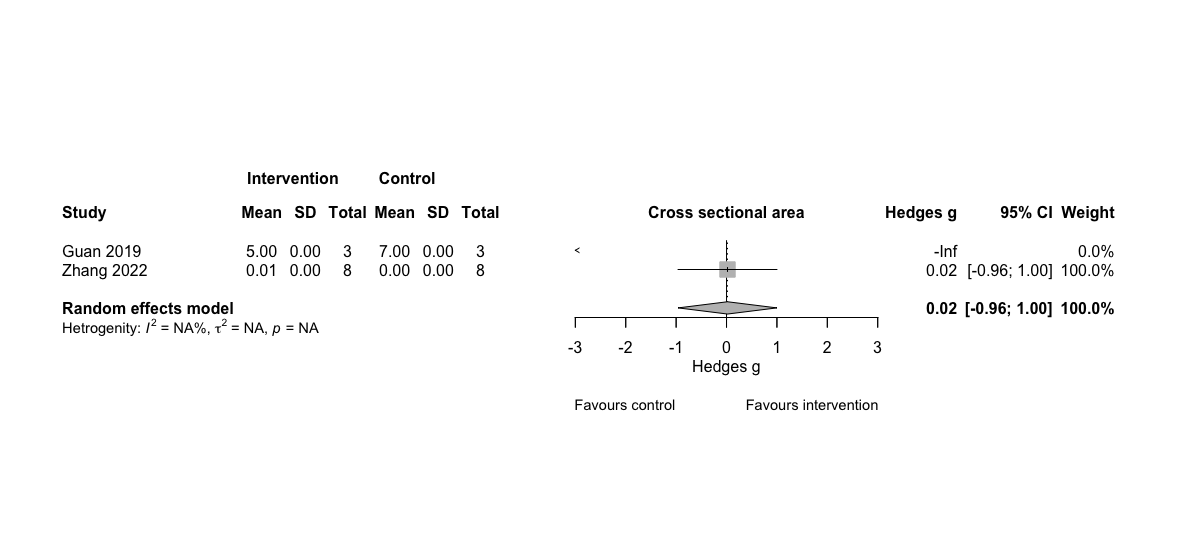 |
| Tensile strength, stress |
| 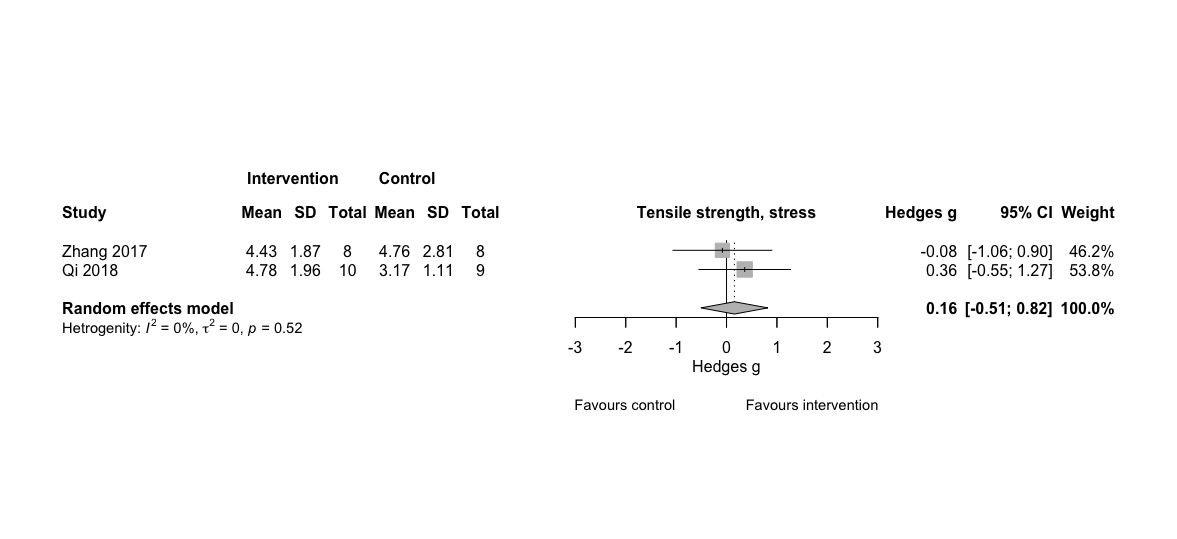 |
| Tensile strength, strain |
| 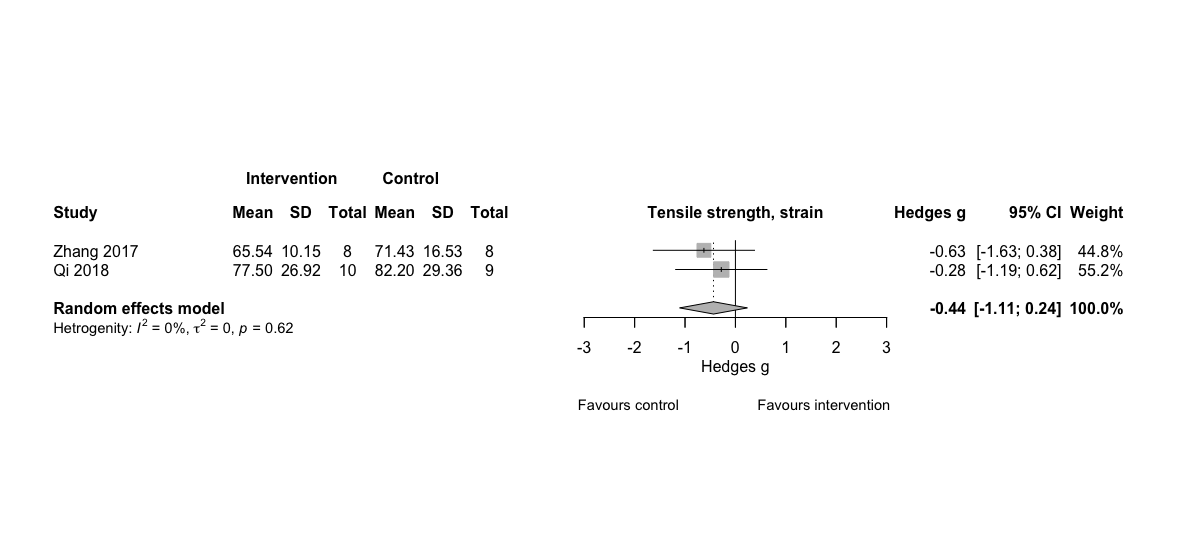 |
| Function |
| Blood flow, Velocity |
| **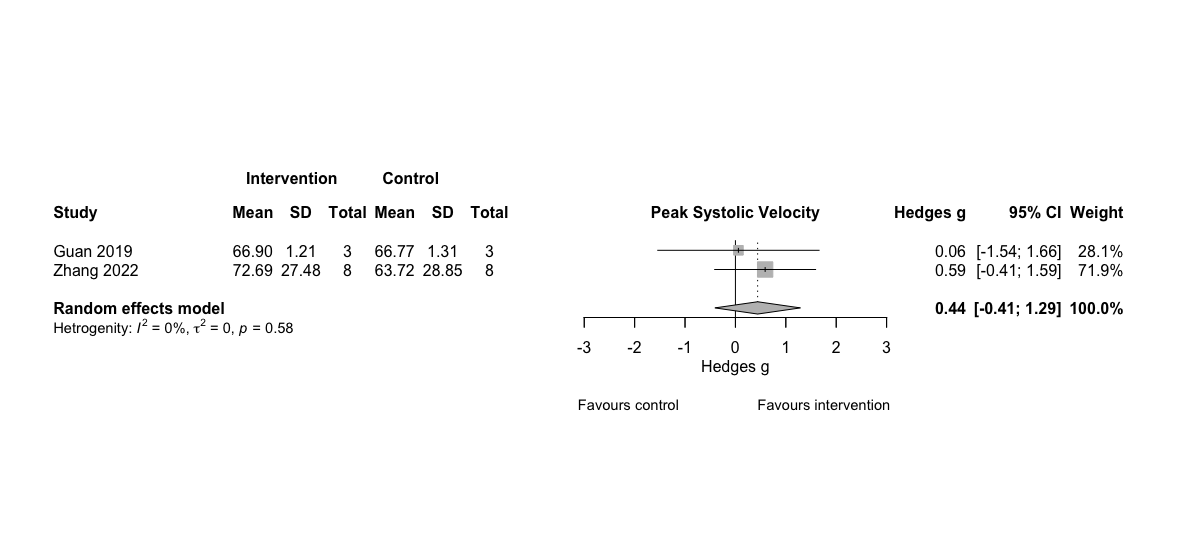** |

Forest plots of the effects of induced vascular pathology versus no induced vascular pathology when treated with cSMT on vascular structure, function, inflammatory markers or mortality (Research Question 3)

| Structure |
| --- |
| Tensile strength, stress |
| 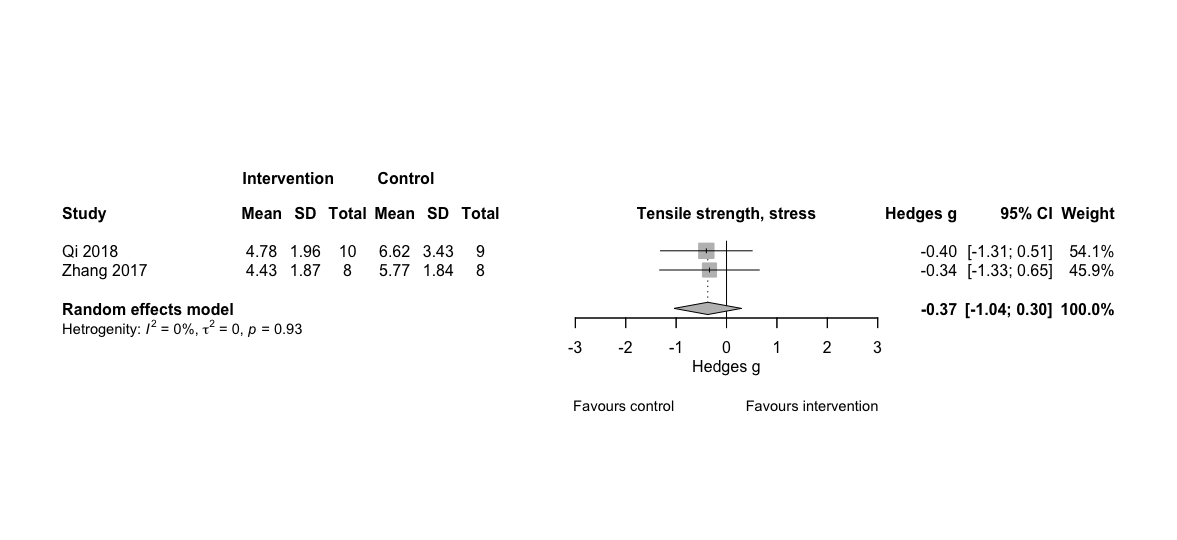 |
| Tensile strength, strain |
| 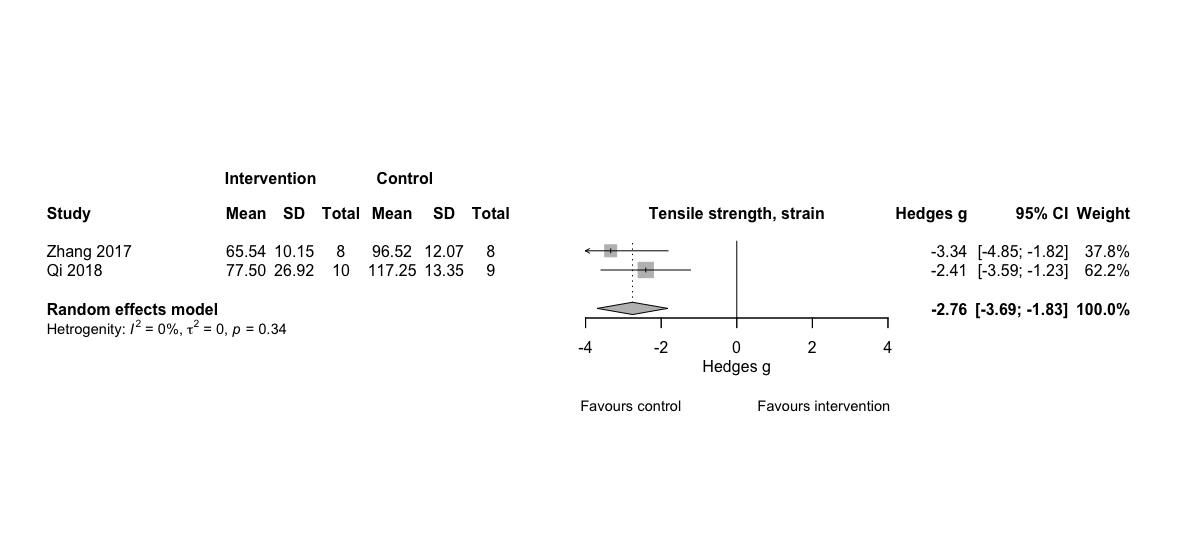 |
